# Histology and Morphology of the Brain Subarachnoid Trabeculae

**DOI:** 10.1155/2015/279814

**Published:** 2015-05-24

**Authors:** Parisa Saboori, Ali Sadegh

**Affiliations:** ^1^Department of Mechanical Engineering, Manhattan College, Manhattan College Parkway, Riverdale, NY 10471, USA; ^2^Department of Mechanical Engineering, City College, City University of New York, 160 Convent Avenue, NY 10031, USA

## Abstract

The interface between the brain and the skull consists of three fibrous tissue layers, dura mater, arachnoid, and pia mater, known as the meninges, and strands of collagen tissues connecting the arachnoid to the pia mater, known as trabeculae. The space between the arachnoid and the pia mater is filled with cerebrospinal fluid which stabilizes the shape and position of the brain during head movements or impacts. The histology and architecture of the subarachnoid space trabeculae in the brain are not well established in the literature. The only recognized fact about the trabeculae is that they are made of collagen fibers surrounded by fibroblast cells and they have pillar- and veil-like structures. In this work the histology and the architecture of the brain trabeculae were studied, via a series of *in vivo* and *in vitro* experiments using cadaveric and animal tissue. In the cadaveric study fluorescence and bright field microscopy were employed while scanning and transmission electron microscopy were used for the animal studies. The results of this study reveal that the trabeculae are collagen based type I, and their architecture is in the form of tree-shaped rods, pillars, and plates and, in some regions, they have a complex network morphology.

## 1. Introduction

**Figure 1:**
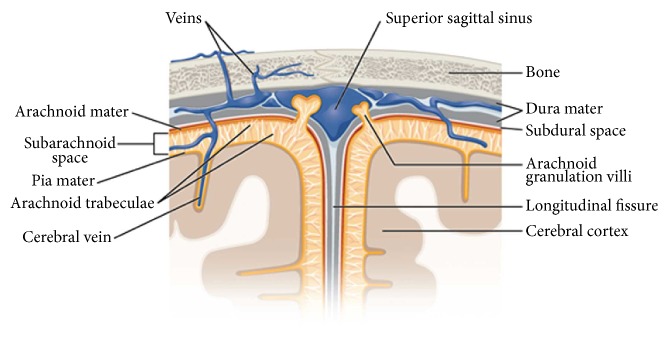
Meningeal layers, the SAS, the pia mater, and the arachnoid. OpenStax College: Anatomy & Physiology, Connexions Web site. http://cnx.org/content/col11496/1.6/, Jun 19, 2013.

Traumatic brain injury (TBI), which is mainly due to vehicular collisions, contact sports, falls, or shock wave blasts from improvised explosive devices (IEDs), is caused by the relative motion between the brain and the skull. Anatomically the interface between the skull and the brain consists of a series of three fibrous tissue layers, dura mater, arachnoid, and pia mater, and arachnoid trabeculae which are strands of collagen tissue (Figure 1). In addition, the space between the arachnoid and pia mater, known as the subarachnoid space (SAS), is filled with cerebrospinal fluid (CSF) which stabilizes the shape and the position of the brain during head movements.

The SAS itself has a complex geometry due to the fact that there is an abundance of trabeculae which stretch from the arachnoid (subdural) to the pia mater. Also, since the pia mater adheres to the surface of the brain and follows all its contours including the folds of the cerebral and cerebellar cortices, the resulting SAS is highly irregular and the associated distribution of CSF within the SAS is very nonuniform. Consequently, this irregular geometry produces a complex CSF flow around the brain, which results in a solid-fluid interaction that damps and stabilizes the movement of the brain when the head is exposed to external loads.

Unfortunately, the complicated geometry of the SAS and trabeculae makes it impossible to model all the details of the region. Thus, in many studies [1–6] the meningeal layers and the subarachnoid region have been simplified as a soft elastic material or, in some cases, as water (i.e., as a soft solid having the bulk modulus of water and a very low shear modulus, as was done by [2, 5, 6]). The shortcoming of these approaches is that the hydraulic damping associated with the fluid solid interaction and the mechanical role of the fibrous trabeculae and the CSF in the subarachnoid space have often been ignored.

This is borne out by the fact that the subarachnoid space (SAS) trabeculae play an important role in damping and reducing the relative movement of the brain with respect to the skull, thereby reducing traumatic brain injuries (TBI), as was shown by Zoghi-Moghadam and Sadegh [7]. While the functionality of the SAS is understood, the architecture, the histology, and biomechanics of this critical region have not been fully investigated. Consequently, in the modeling of the head, previous investigators have oversimplified this important region, and these simplifications could lead to inaccurate results in a finite element analysis of the brain.

With regard to material properties various studies [4, 6, 8] have reported a wide range of elastic moduli for the trabeculae that are up to three orders of magnitudes different in value, (from* E* = 59.8 × 10^3^ Pa, Jin et al. [8], to* E* = 21.5 × 10^6^ Pa, Zhang et al. [6]), which brings into question the usefulness of these values. However, several validated finite element models (e.g., [9]) have reported a value of 1150 Pa as a realistic material property for the SAS.

In addition, there have been a few experimental studies associated with the architecture of the SAS, with one experimental study by Alcolado et al. [10], which involved examining the calcification in the human choroid plexus, meningiomas, and pineal gland of 20 postmortem brains and one biopsy. The result of that work was compared with calcification in psammoma bodies in a normal arachnoid, and there it was concluded that psammoma bodies in the choroid plexus form by a process of dystrophic calcification associated with arachnoid cells and collagen fibers. In contrast, Frederickson [11] used electron microscopy to study the subdural region within the cranial meninges in guinea pigs, where attention was paid to the fine structure of the arachnoid membrane, dura mater, inner surface of the dura, and outer surface of the arachnoid. This study revealed that the subdural space was not observed in the guinea pig, and it was also concluded that the reason the intermediate cells are located in the light cell layer, next to the dark arachnoid cells, is because of a greater complement of rough endoplasmic reticulum. In addition, the histology of the trabeculae in the optical nerves was studied by Killer et al. [12], and this work was based on 12 optic nerves harvested from nine subjects. The samples were examined within seven hours after death, following qualified consent for necropsy. Scanning electron microscopy (SEM) and transmission electron microscopy (TEM) were used to study the anatomy and arrangement of trabeculae within the optic nerves and they were described as having pillar-, septa-, and platelike structures similar to the trabeculae found in a subarachnoid space. The conclusion from this study was that the human optic nerve is not a homogeneous medium and that the SAS is regionally different, particularly in the area closer to the canalicular portion where the trabeculae are more oriented and shaped like pillars. In a recent study by Scott and Coats [13], optical coherence tomography (OCT) Imaging was used to determine the density and regional variability of arachnoid trabeculae within the SAS. From this study it was concluded that more investigation is needed to study the architecture of the SAS using OCT imaging techniques.

The goal of this present study was therefore to investigate the histology and morphology of the SAS of the brain and in particular the SAS trabeculae, which is needed for sophisticated and accurate modeling of TBI. Specifically, in this paper, the histology and the architecture of the brain trabeculae are presented via cadaveric and animal experimental studies. In the first experimental study of the brain a histological sectioning with florescent and bright field illumination was done. In the second set of experimental studies scanning and transmission electron microscopy were used.

## 2. Material and Methods

### 2.1. Cadaveric Experimental Studies

The first set of experiments designed to examine the histology and architecture of the SAS was done, by using fluorescence and bright field microscopy, to determine the structure of the SAS associated with a cadaver brain.

**Figure 2:**
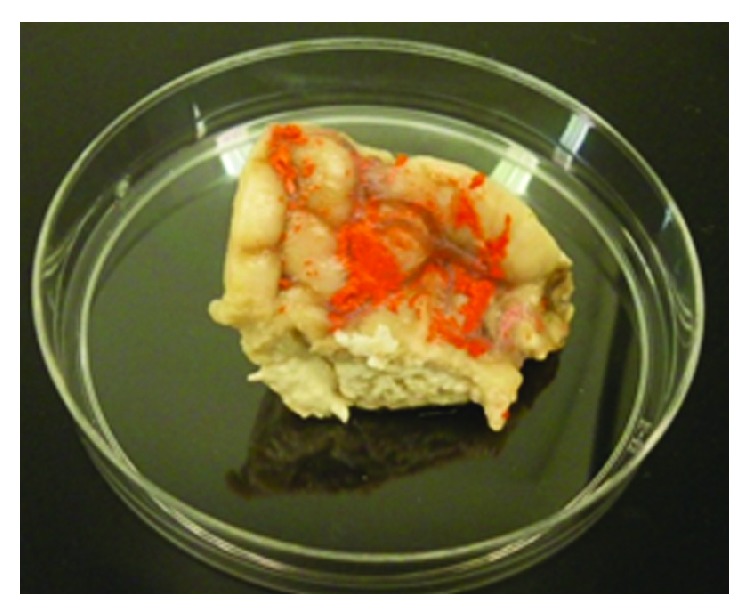
The brain tissue with Microfil fixed in the formalin.

In this experiment an image of the trabecular architecture of a cadaver was acquired using florescent and bright field microscopy. However, since the cadaveric human brain was already fixed by formaldehyde, the arachnoid had been collapsed onto the pia mater and the CSF had been drained. Several techniques were employed to separate the arachnoid from the pia mater and to recreate and restore the subarachnoid space, which is approximately 2 to 3 mm wide in a human head. The techniques involved several steps that included confining a cortical region of the brain and the injection of Microfil (a silicone rubber injection compound) from Flowtech, Inc., into the region between the two layers of the arachnoid and the pia mater. The Microfil solidified quickly and kept the two layers separated. To inject the Microfil solution into the SAS a fixture was designed to confine the Microfil within the cortex, where the fixture consisted of a clear tube with a key-way on one side to allow for the insertion of the syringe needle. Extreme care was taken during this process since it was necessary to ensure that the solution was injected exactly between the pia mater and the arachnoid. The viscosity of the fluid was also an important factor because if the Microfil that was mixed with the solidifier was too thick it was not possible to inject it between the two layers using a small needle; however, if it were diluted too much it would then just be drained out from between the neighboring cortexes and would not cause the subarachnoid space to open. Several tissue samples from different regions of the cadaver's brain were prepared using this technique.  Figure 2 shows the Microfil injected between the arachnoid and the pia mater to separate and rebuild the SAS of the brain tissue. The samples were then stained using hematoxylin-eosin staining protocol and sliced using a vibratome. It should be noted that since the tissue was already fixed in the formaldehyde the Microfil did not survive the staining procedure and it was washed out during the sample preparation. However, it was possible to see the trabeculae structure and make a distinction between the pia mater and the arachnoid mater.

### 2.2. Animal Experimental Studies

In addition to the cadaver work, scanning electron microscopy (SEM) and transmission electron microscopy (TEM) were used to study the morphology of trabeculae and to obtain a better understanding of the density and the configuration of the trabeculae network in a rat's brain. A rat was used since it has been shown that there is a similarity in the morphology of the trabeculae of rats and humans [10, 11, 13]. This work was approved by the City College of the City University of New York Institutional Research Board.

#### 2.2.1. Scanning Electron Microscopy (SEM)

To investigate the histology and the architecture of the SAS trabeculae* in vitro*, experiments were performed using Sprague-Dawley rats that weighted 250–300 g and were 2 to 3 months old. The objective of this experiment was to fix and solidify the subarachnoid space and the trabeculae while the animal was alive. The rats were anesthetized with pentobarbital sodium (PBS) given subcutaneously (80–100 mg/kg body weight for initial anesthetization and 30 mg/kg body weight for maintenance as needed) and kept warm on a heating pad. A prefixative solution of PBS followed by the fixative solution (glutaraldehyde and formaldehyde) was injected through the cut ventricle into the ascending aorta of the rat to allow the blood vessels of the SAS to be solidified. The animal was sacrificed and samples of the brain tissue were prepared for the scanning and transmission electron microscopy. The head was cut, the skull was carefully dissected, and the brain was extracted along with the dura mater. The samples were kept in a glutaraldehyde 5% solution overnight to ensure the tissue was cross-linked and it was completely fixed. The regular protocol of biological sample preparation was used with some adaptations for these types of samples. Cerebral sections were obtained by cutting along a sagittal plane. The samples were then cleaned of the glutaraldehyde solution under a hood, and the sections needed for visualization under the SEM were obtained and washed in distilled water (ddH_2_O). Care was taken to keep the samples constantly wet, and after 5 minutes the samples were rinsed with fresh ddH_2_O. In total, the samples were washed 5 times with ddH_2_O, each time for 5 minutes. At the end of this step, none of the glutaraldehyde solution remained in the samples. Next, the samples were placed in a basket and the ddH_2_O was washed out from the samples by using alcohol at different concentrations.

The basket was then placed in a tube and 30 mL of alcohol at 25%, 50%, 70%, 80%, 90%, and 100% concentrations was added consecutively to the tube for 5–10 minutes each time in a second step to further guarantee that no water remained within the sample. The tube was shaken from time to time to ensure a better penetration of the alcohol within the tissues. The Critical Point Drying (CPD) technique was also used by immersing the samples in a chamber that was then placed in liquid CO_2_, rinsing them 7 times for 5–10 minutes. After the alcohol was washed out, the samples were dried, and the temperature of the chamber was raised to 40°C, letting the CO_2_ change from its liquid phase to a dry gas phase. Finally, the samples were coated with gold particles just prior to observing them with the SEM.

#### 2.2.2. Transmission Electron Microscopy (TEM)

The tissue samples for the TEM study were obtained from the same batch of brain tissue prepared for use with the SEM; however, in this process the specimens had to be only 1 or 2 mm in thickness; therefore it was necessary to choose and cut the tissue samples from a specific location of the brain due to the small physical size of the specimen. Consequently, extracting the tissue from the area surrounding the superior sagittal sinus by cutting the brain along a plane parallel and next to the sagittal plane on both sides of the sinus was decided. The sections were then shortened to be about 1 mm from the top of the brain and were cut into approximately 1 mm sized pieces. The samples were subsequently immersed in acrolein for an hour followed by a secondary fixative using an osmium tetroxide 2% solution, and then they were washed with ddH_2_O three times for 5 minutes. The samples were dehydrated and rinsed twice in propylene oxide for 5 minutes. For each concentration the jar containing the resin and the samples was placed on a shaker for three hours. Eventually each piece was transferred to the bottom of a triangle-shaped beam capsule with a pipette and then each capsule was filled up to three-quarters of the way with resin at 100% concentration. Finally, the capsules were placed in an oven at 60°C to allow the resin to solidify.

The block sample was transferred to the microtome and its position was adjusted with respect to the diamond knife. The thickness of the sections was set to 100 nm. Once enough sections had been obtained, a moist eyelash was used to create groups of six pieces, and they were transferred onto the shiny side of the small round grids. Subsequently, the samples were stained with uranyl acetate and Reynolds lead citrate and studied using a Philips CM-12 transmission electron microscope at an accelerating voltage of 80 kV.

## 3. Result

### 3.1. The Fluorescence and Bright Field Result

**Figure 3:**
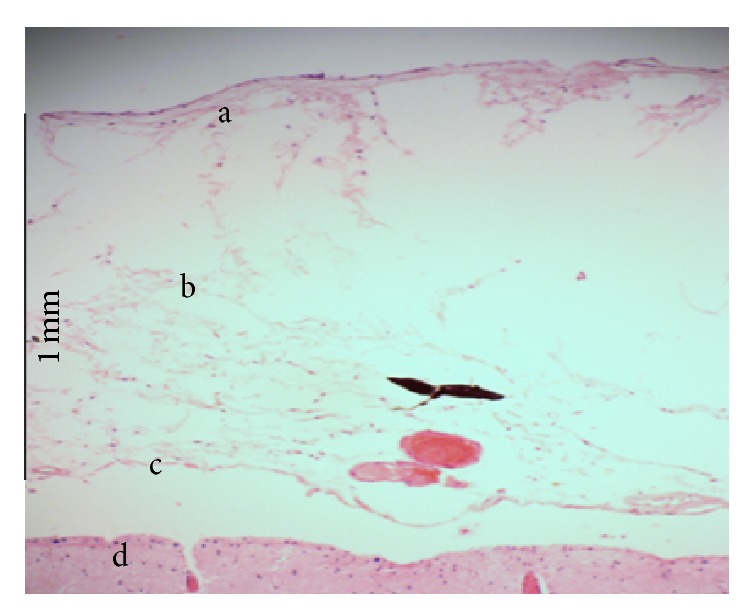
Light microscopic view of subarachnoid, (a) the arachnoid mater, (b) SAS, (c) the pia mater, and (d) the brain.

**Figure 4:**
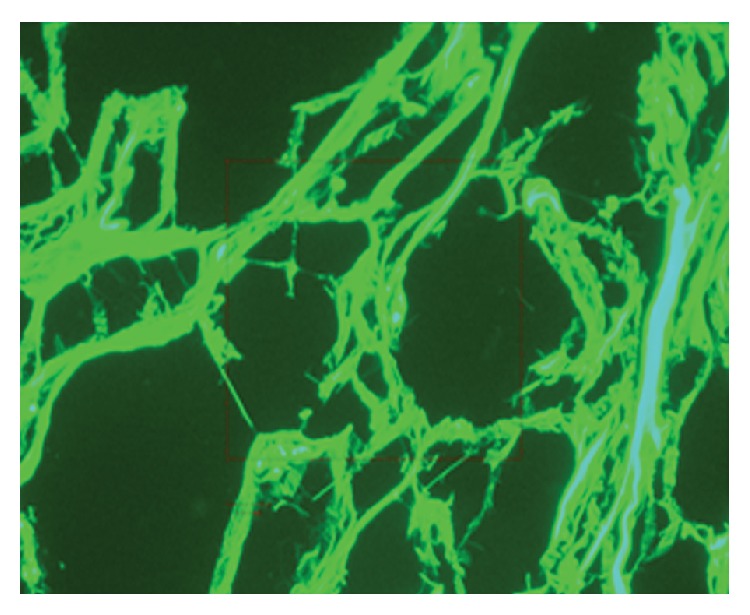
Trabeculae of cadaveric brain tissue under the fluorescent light.

The bright field microscopy image of the subarachnoid space is shown in Figure 3, where the arachnoid and the pia mater are identified. The figure also shows that there is a gap between the pia mater and the brain which is caused by the injection of the Microfil. The tissue studied under the fluorescence light microscopy is shown in Figure 4, and here collagen tissues can be clearly seen. From these initial experiments it was determined that the subarachnoid space collapses easily in the absence of CSF, and injection of rubber silicon may rupture the trabeculae.

### 3.2. SEM Results

**Figure 5:**
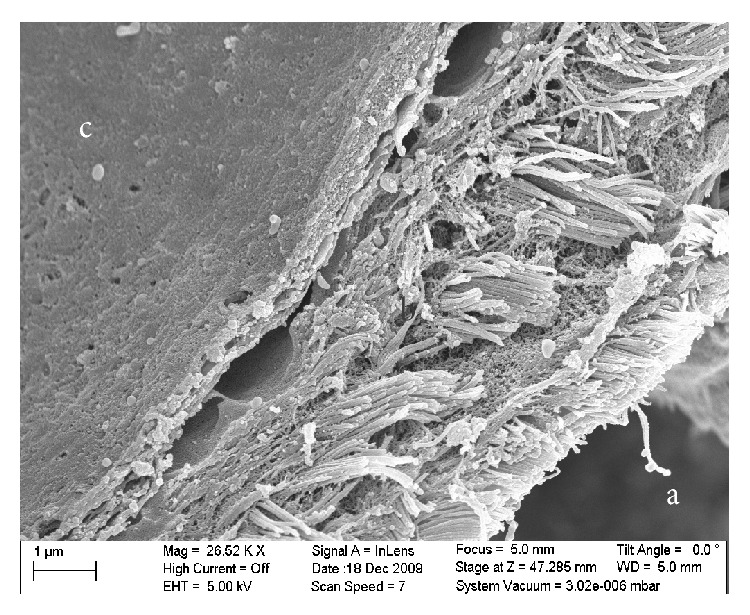
SEM micrograph of the dura and arachnoid layer in the rat brain: (a) the brain side, (b) the SAS, and (c) the dura mater.

**Figure 6:**
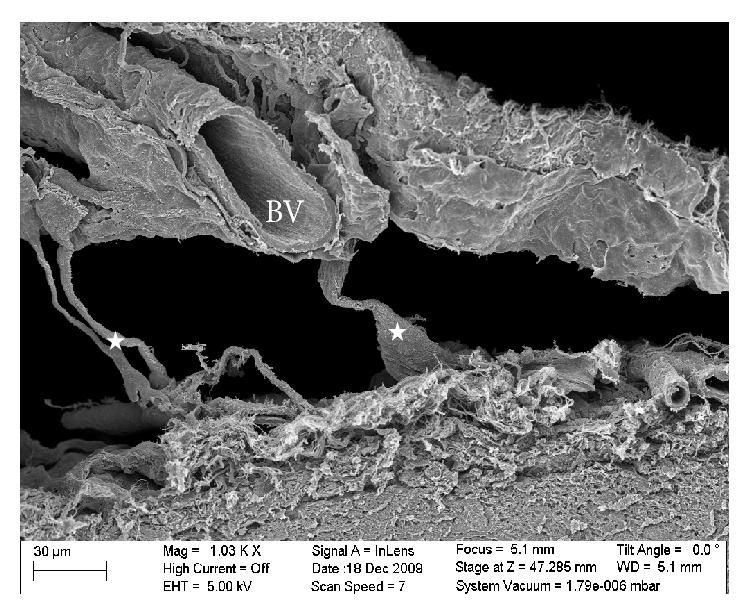
SEM image of the SAS in the rat with some trabecula (star) surrounding a blood vessel (BV).

**Figure 7:**
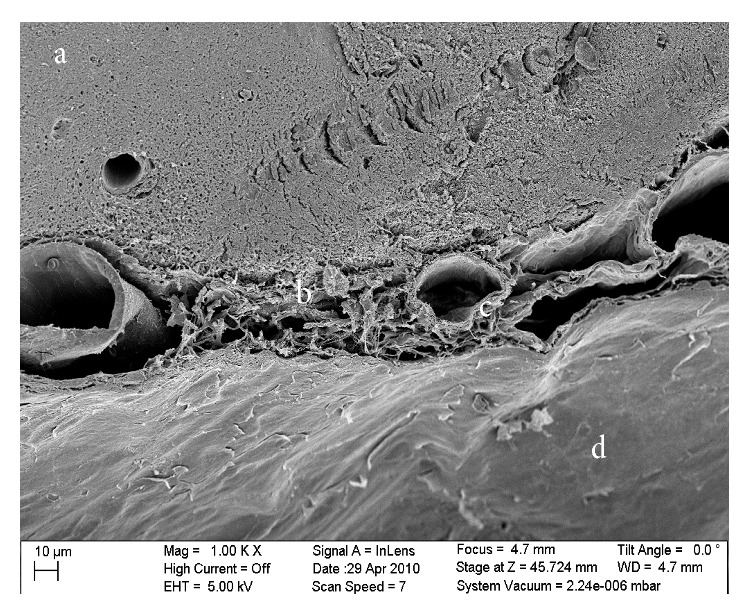
The SEM image of the rat's brain: (a) the brain, (b) the veil-like network of trabeculae, (c) the blood vessel in the SAS, and (d) the dura mater.

**Figure 8:**
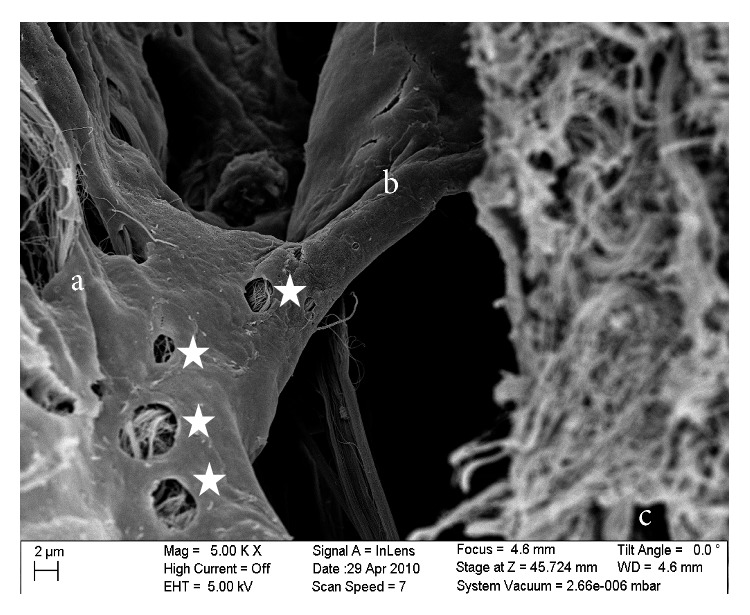
SEM image of a platelike trabecula showing permeable characteristics (stars): (a) the arachnoid mater, (b) the plate trabecula, and (c) the pia mater.

**Figure 9:**
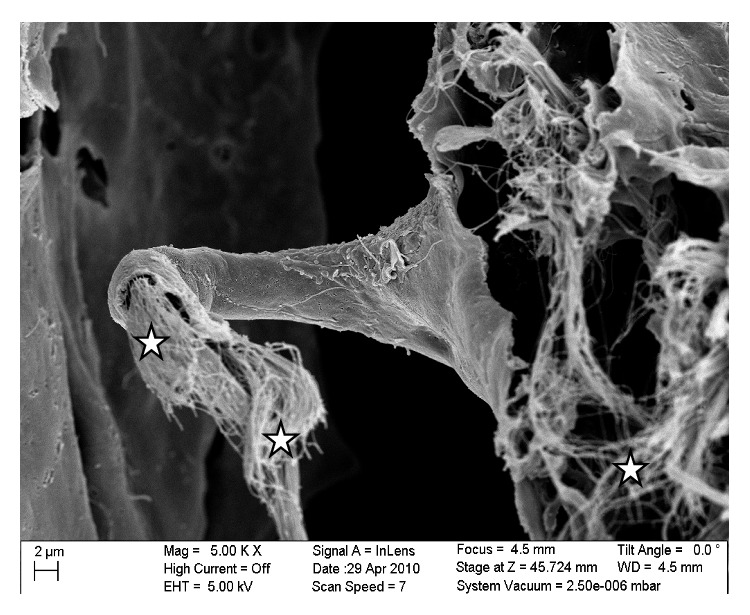
Image of the collagen fibrils (star) that constitute the internal structure of a trabecula.

The results obtained from the SEM showed that the SAS was almost fully open and the blood vessels were extremely well preserved, as shown in Figures 6, 8, and 9. These images allowed the different layers of the meninges, the SAS (Figure 5), the trabecula (Figure 6), and their fine structures (Figure 8) to be clearly seen. The high resolution of the SEM permitted the structure of the trabecula to be seen down to the tissue level. Figures 5 to 9 reveal the morphology and architecture of the SAS.

Specifically, Figure 8 is a close-up of an SEM image of a trabecula that has a platelike shape that spans the arachnoid and pia mater. The voids in the trabecula show the permeable characteristics of a trabecula itself with the voids being approximately 0.5–3 *μ*m in size.  Figure 9 shows a further close-up of a trabecula and in this image it is clearly seen that its internal structure consists of collagen fibrils. In addition, Figure 8, along with Figure 9, shows the tree-shaped structure of a trabecula where the stem is on the pia mater and the branches merge into the arachnoid mater.  Figure 9 is a close-up of a different area of the SAS where the trabecula has a veil-like network structure. This type of structure is seen in the areas where the trabeculae are denser, such as between blood vessels.

The SAS appeared to have a more complicated morphology than is presented in much of the literature [8, 14–16]. Instead of being merely rods connecting the pia mater to the arachnoid layer, the trabecula displayed various shapes and organizations. In this work trabeculae were seen to have a variety of forms: simple or branched rods, tree shapes, pillars, plates, or complex networks. However, it was clear that the basic building blocks for the components that make up the SAS were collagen fibrils. Those fibrils were found everywhere within the SAS (Figures 8 and 9) and provided the tissue with its structural and permeable characteristics (Figure 8). The SEM images and figures shown in Killer et al. [12], which were from the SAS of the human optic nerve, confirmed these results (i.e., both results reveal that SAS trabeculae exhibit the same kind of morphology and shape); it is also important to note that these similarities exist between two different species of brain tissue (i.e., human and rat).

### 3.3. TEM Results

**Figure 10:**
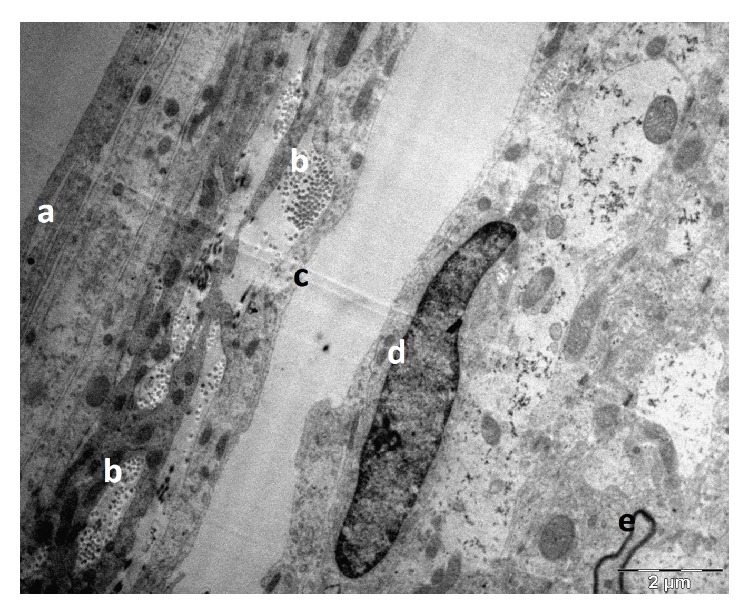
TEM section of the SAS in the rat's brain: (a) the arachnoid mater, (b) collagen fibrils cross section, (c) the SAS, (d) nucleus in pia mater, and (e) the axon in the gray mater.

**Figure 11:**
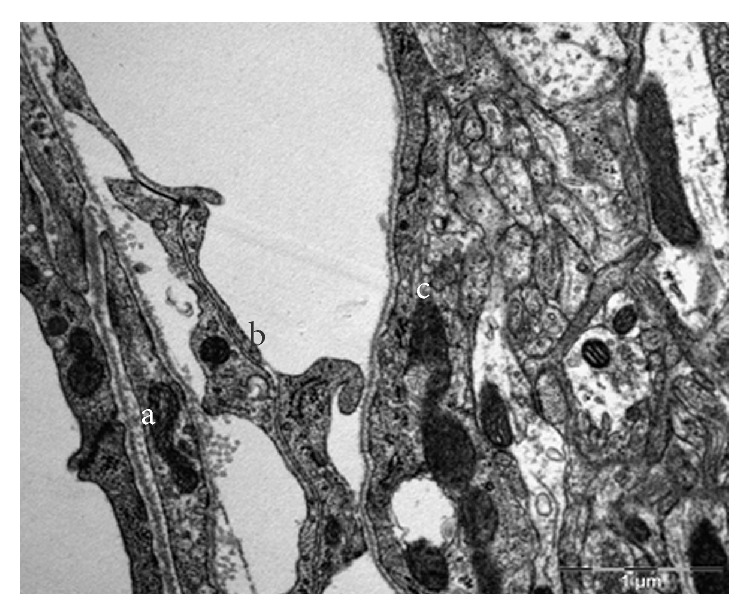
TEM section of the SAS in the rat: (a) the arachnoid mater, (b) the SAS architecture (trabeculae), and (c) the pia mater.

**Figure 12:**
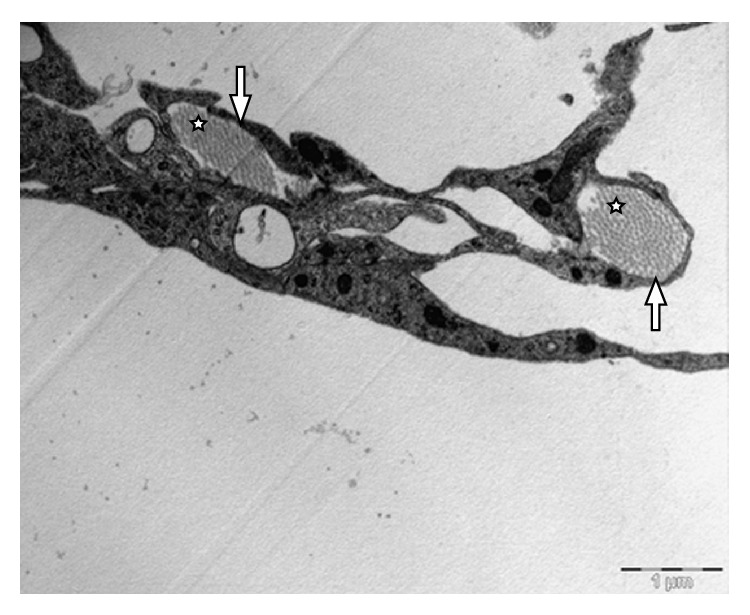
Bundle of collagen fibrils (stars) surrounded by fibroblasts (arrows).

**Figure 13:**
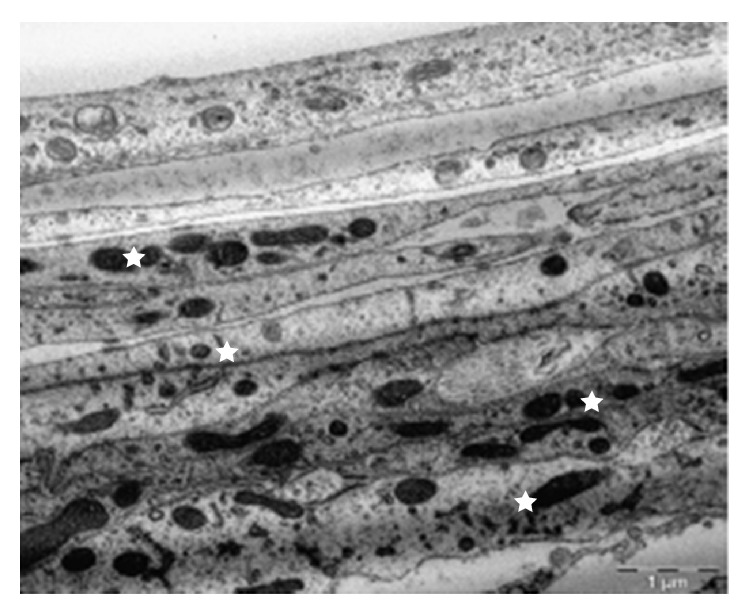
Layers of fibroblast cells in the arachnoid. The flat cells on the arachnoid side were well defined and arranged in layers (stars).

**Figure 14:**
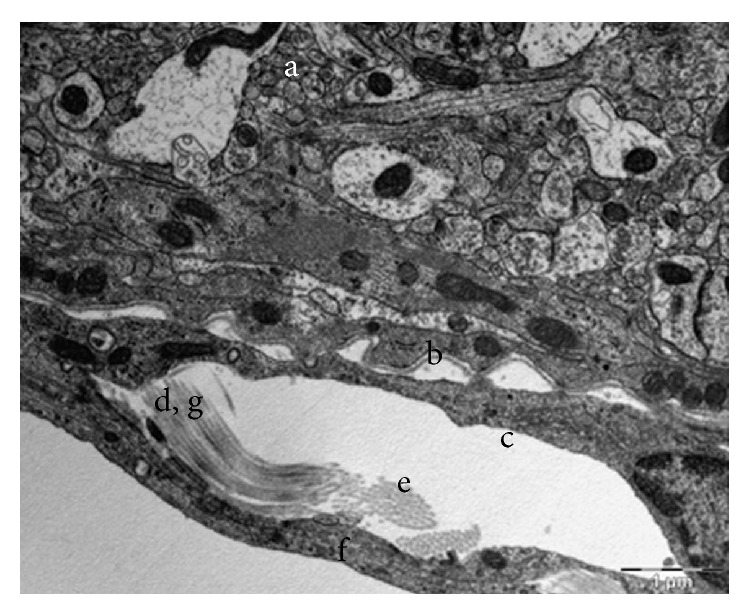
Longitudinal and cross section of collagen fibers in the arachnoid side of the SAS: (a) the brain, (b) the basal lamina, (c) the pia mater, (d) longitudinal section of collagen fibers, (e) cross section of collagen fibers, (f) the arachnoid mater, and (g) periodicity.

The TEM images allowed the cellular and subcellular structure of the SAS to be observed. In comparison to the SEM, the TEM images allowed different cell layers to be clearly identified; however, the separation between the brain and the SAS was not as clear as in the SEM case; therefore the myelin sheath of the neurons was taken as a reference to locate the brain. Figures 10 to 13 show different orientation of the SAS in the rat's brain, while Figure 14 shows the longitudinal and cross section images of the collagen fibers in the SAS. The thickness of individual fibers, the general appearance of the bundles, and the regular periodicity of the structure observed in Figure 14 confirmed that the trabeculae are type I collagen, Abraham [17]. However the periodicity is better seen in samples that are fixed with the addition of 1% phosphotungstic acid to the glutaraldehyde fixative. In addition, the brain, the basal lamina, the pia mater, the longitudinal section of collagen fibers, the cross section of collagen fibers, and the arachnoid mater are also clearly identified. Specifically, Figure 10 shows the TEM section of the SAS in the rat's brain where the arachnoid mater, collagen fibril cross sections, the SAS, the cross section of a trabecula, and the axon in the gray mater can all be clearly identified; these are denoted by using letters (a) through (e), respectively.  Figure 12 is a close-up of the TEM image showing a bundle of collagen fibrils surrounded by fibroblasts, and Figure 13 shows layers of fibroblast cells in the arachnoid region (note that the flat cells on the arachnoid side were well defined and arranged in layers).

The results from the TEM provided good information about the cellular arrangement within the SAS. Specifically, the flat cells on the arachnoid side of the SAS were well defined and arranged in layers as shown in Figure 14, whereas in the brain it was difficult to define the contour of some cells except for the axons of the neurons which were surrounded by a dark layer. The blood vessels also helped to locate the SAS. As expected the blood vessels had not collapsed, and the gap surrounding them indicated that the SAS had also not collapsed. It was observed that the collagen fibrils of trabeculae were arranged in groups surrounded by fibroblastic cells. These bundles could be found at different locations and were lining the fibroblasts of the arachnoid layer, inserted within the arachnoid layer, or linked to the blood vessels via a fibroblast. In the TEM the fibrils were observed to have two orientations, transverse (Figure 12) and lateral (Figure 14), with the transverse orientation being seen when the fibrils appeared as dots (Figure 12) and the lateral orientation being seen when the fibrils appeared as forming long thin light and dark stripes (Figure 14). This confirmed the rodlike nature of the trabeculae as observed from the SEM images.

In addition, when these bands were examined using high magnification, they were seen to be composed of an alternating collection of light and dark strips, and it is known that such periodicity is a characteristic of the type of collagen that forms the fibrils. It was also observed that the fibril bundles appeared to have different compositions with some bundles being full of fibrils tightly packed whereas other bundles were half or less full of fibrils. The reason for the inhomogeneity among bundles appeared to be that the arachnoid did not need to be as hard as some other structures containing collagen in the body. The collagen fibrils forming the trabeculae have a filler role rather than a support role.

In the SEM results (Figures 5, 6, and 7), the dura mater and the arachnoid mater appeared to possess very densely packed structures; therefore it was assumed that those layers would be impermeable. However, in the TEM results (Figure 14), it was noted that some liquid was located between the cells deep down within the arachnoid layer. It was therefore concluded that this layer must be permeable to some degree and that some fluid can flow between the fibroblasts at some locations.

It should also be noted that the TEM required a much longer preparation process than did the SEM. The preparation of samples had more steps, and many of those steps were critical for the cleanliness of the sections; however the benefit was that the TEM images provided information about the cellular content of the SAS that was lacking in the SEM. Specifically, it was found from these results that the arachnoid layers were made of fibroblasts and not only of collagen fibrils, that these cells formed a permeable layer, that the pia mater was represented by a single layer of fibroblast cells covering the brain, and that the collagen fibrils were arranged in bundles and scattered within the SAS at the border within the arachnoid layer and around the endothelial sheet of the blood vessels.

## 4. Discussion

A wide range of material properties for the SAS, of up to three orders of magnitude different, have been reported in the literature. In a previous study performed by Saboori [18], the transmission of an external impact load to the head, which passes through the SAS and into the brain, was investigated, and the optimum mechanical properties of the SAS trabeculae were identified. The selected material property was based on the validation of the model with the experimental results of Sabet et al. [19] and Feng et al. [20] and it was determined that the SAS material properties used by previous investigators were too stiff and could lead to unreliable finite element analysis results. It was also concluded that the material properties of the trabeculae should be simulated using tension-only elements, since the trabeculae buckle with a minimal compressive load, Zoghi-Moghadam and Sadegh [7]. Because of these uncertainties, this study was designed to identify the histology and architecture of the SAS and, in particular, the structure of the trabeculae. This was accomplished via two experimental studies that included cadaver and animal studies. In the cadaver study a basic understanding of the morphology of the SAS trabeculae was obtained, while in the animal study more detailed information was collected by using scanning electron microscopy (SEM) and transmission electron microscopy (TEM).

With regard to the arachnoid mater, the experimental studies revealed that it was composed of about ten layers of fibroblasts cells joined together via tight junctions. In contrast the pia mater appeared to be much thinner than the arachnoid layer since it was composed of only one layer of fibroblasts. Also within the cells of the arachnoid, some collagen fibril bundles could be observed, with the fibrils being produced by the fibroblasts, and thereby provided a structural support to the arachnoid layer, in addition to the support provided by the fibroblasts themselves. The arachnoid layer appeared to be permeable in the TEM results, and some fluid was observed in the spaces between the fibroblasts. At the junction of the arachnoid layer with the SAS the trabeculae branch out to meet the arachnoid mater at some locations and form a descending trabecula from the arachnoid to the pia mater in tree-shaped structures.

In the case of the trabeculae, the experimental studies revealed that the architecture of the trabecular could be quite complex with individual structures being like tree-shaped rods, pillars, and plates and in some cases having an intricate veil-like geometry. Structurally, the trabeculae were found to consist mainly of bundles of collagen fibrils wrapped together by fibroblast cells, with the fibroblasts being found in all connective tissues. In addition, the trabecula were found to be surrounded by an extracellular matrix (ECM) with a thickness of between 50 and 200 nm and composed of collagen fibers, proteoglycans, laminins, and fibronectins.

**Figure 15:**
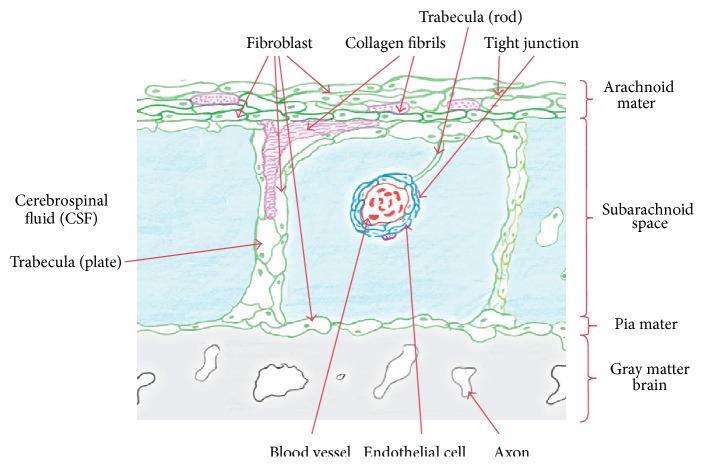
Cartoon sketch of the SAS in a rat's brain.

In addition to the rods, plates, and tree-shaped architecture, it was observed that in some regions complex networks of more randomly oriented trabeculae were found in the SAS. This network architecture was mainly located in the vicinity of the blood vessels and was very complex and inhomogeneous. These trabeculae networks were composed of fibroblasts, collagen fibrils, and extracellular matrix. Some holes/cavities were also seen to exist in the networks. These cavities facilitated the flow of cerebrospinal fluid around the brain. The results of this experimental study were compared and validated with regard to a study by Killer et al. [12]. While these results are for a rat brain it is believed that a human brain would have the same or a similar trabecular architecture. From SEM and TEM results it was then possible to create a cartoon sketch of the SAS in the rat showing all of the salient features (Figure 15).

## 5. Conclusion

It was concluded from this work that the trabeculae are collagen based Type I, as was found by Abraham [17] and van der Rest and Garrone [21]. It was also observed that the arachnoid mater was composed of approximately ten layers of fibroblasts cells joined together by tight junctions, with these molecules being connected together in a 3D network to provide a cell-to-cell interconnection, a cell-substrate interconnection, or a mechanical support for the tissue and with the fibroblasts being connected to the blood vessels present within the SAS and the pia mater and to the arachnoid mater via specific junctions. The results of this experimental work therefore provide a basis for a better understanding of these tissues and their fine structures and provide data for accurate modeling of the SAS layer. Those networks were mainly located in the vicinity of the blood vessels and were very complex and inhomogeneous. The images reveal that the meshes of trabeculae are actually composed of fibroblasts, collagen fibrils, and other extracellular matrix constituents. Some spaces and voids could also be observed in the networks which facilitate the flow of cerebrospinal fluid (CSF). The trabecular structures were attached to both the arachnoid and pia sides of the SAS. Nevertheless the pia mater appeared to be much thinner than the arachnoid layer as it was composed of only one layer of fibroblasts.
